# Utilization of a chicken embryo membrane model for evaluation of embryonic vascular toxicity of *Dorema ammoniacum*

**Published:** 2020

**Authors:** Hadi Tavakkoli, Amin Derakhshanfar, Javad Moayedi, Ali Poostforoosh Fard

**Affiliations:** 1 *Department of Clinical Science, School of Veterinary Medicine, Shahid Bahonar University of Kerman, Kerman, Iran*; 2 *Diagnostic Laboratory Sciences and Technology Research Center, School of Paramedical Sciences, Shiraz University of Medical Sciences, Shiraz, Iran*; 3 *Center of Comparative and Experimental Medicine, Shiraz University of Medical Sciences, Shiraz, Iran *; 4 *Executive Secretary of Medical Ethics Committee, Vice-Chancellery of Research and Technology, Shiraz University of Medical Sciences, Shiraz, Iran*

**Keywords:** Dorema ammoniacum, Embryo, Fetus, Pathology, Angiogenesis, VEGF-A

## Abstract

**Objective:**

Extensive research has been done to assess the efficacy of herbs for treating different disorders. *Dorema ammoniacum* (*D. ammoniacum*) is used in folk medicines for various goals. The application of herbs in medicine is accompanied by harmful effects. Chick embryo is considered a suitable model for assessing drugs toxicity. The present study aimed to evaluate the changes in vasculature in chick’s extra-embryonic membrane following *D. ammoniacum *treatment. Alterations in molecular pathways associated with early embryonic angiogenesis such as vascular endothelial growth factor A (*VEGF-A*) were also evaluated.

**Materials and Methods:**

Fertile chicken (Ross 308) eggs were allocated into three similar groups; sham, control and *D. ammoniacum *groups; in *D. ammoniacum *group, eggs were inoculated with plant’s extract at doses of 50 or 100 mg per kg egg-weight.

**Results:**

Analysis of the extra-embryonic membrane vasculature revealed that *D. ammoniacum *extract decreases some vascular parameters such as vessels area, total vessels length, vascular branch and increases lacunarity. This herb’s vascular toxicity was in a dose-dependent manner. Down-regulation of the expression of *VEGF-A* was also seen in the extract-treated extra-embryonic membrane.

**Conclusion:**

Vascular toxicity of *D. ammoniacum *was confirmed by data presented in this paper. We conclude that alteration of vascular parameters and gene expression might finally lead to embryo malformation due to* D. ammoniacum *consumption. Therefore, the use of this herb must be limited during the fetal growth period especially at doses higher than 50 mg per kg.

## Introduction

Normal development of the fetus is an important concern and compounds that alter genes expression and vascular network could affect this development (Chaiworapongsa et al., 2016 ▶; Chappell and David, 2016 ▶; Popova et al., 2016 ▶) . Consumption of some herbs during gestation is associated with the risk of fetal defects (Izzo et al., 2016 ▶; Feng and Yang, 2017 ▶; Rouhi-Boroujeni et al., 2017 ▶) . Herbs may also induce vascular injury and affect the embryonic growth. Such effects were described for some herbs in several experiments (Sreekanth et al., 2006 ▶; Dunnick and Nyska, 2013 ▶; Kwan et al., 2014 ▶) . Angiogenesis is described as the formation of new vessels from preexisting vessels and it is considered an important step in normal embryo development (Demir et al., 2007 ▶) . Various factors are recognized to be crucial for the regulation of this process. The most important one is the vascular endothelial growth factor A (*VEGF-A*) that is secreted by specific cells including ectodermal and mesenchymal cells (Liebner et al., 2011 ▶; Shibuya, 2013 ▶; Ferrara and Adamis, 2016 ▶) . Assessment of the adverse effects of drugs on embryo requires the use of a preclinical model. The extra-embryonic membrane (EEM) of chick is used for evaluating the activity of anti-angiogenic compounds (Nowak-Sliwinska et al., 2014 ▶; Ribatti, 2017 ▶) . The EEM vasculature of the chick contains too many vessels, that branch progressively during embryonic growth (Gonzalez-Crussi, 1971 ▶) .

Herbal plants have significant roles in health care system of the industrialized societies (Amiri and Joharchi, 2013 ▶; Somani et al., 2015 ▶; Amiri and Joharchi, 2016 ▶; Mahdavi et al., 2016 ▶) . *Dorema ammoniacum* (*D. ammoniacum*) is used in folk medicine for treating various disorders. Medicinal properties reported for different parts of the plant include antibacterial, antifungal, carminative, stimulant, diaphoresis, diuresis, and anti-spasmodic activity (Yousefzadi et al., 2011 ▶; Motevalian et al., 2017 ▶) . Currently, it is used in human medicine with anti-spasmodic activity. as Also, it is used against bronchitis and sever coughs (Paparozzi, 2005 ▶) . In spite of increasing consumption of various compounds of *D. ammoniacum*, little had been known about the toxic activity of this herb on the vascular plexus of the embryo. Furthermore, the mechanisms by which it affects vascular genesis and expansion have not been described exactly. The current study performed to answer the bellow questions:

- Does *D. ammoniacum *change the early growth of the EEM-vessels?
- Does *D. ammoniacum *change *VEGF-A* expression in EEM vessels?

To respond these questions, a chick embryo model was applied. Analytical software was also used to evaluate the vessel plexus of the embryo’s EEM for determination of *D. ammoniacum* anti-angiogenic activity. Lastly, the results of the real-time PCR assay were employed to confirm the effect of the herb on the expression of the gene that is related to vascular formation.

## Materials and Methods

This experimental study was performed to evaluate the alteration of vascular branching pattern in the chick’s EMM following *D. ammoniacum *treatment. This study evaluated A) Effect of *D. ammoniacum* resin-extract on embryonic angiogenesis and B) Effect of *D. ammoniacum* resin-extract on the expression of *VEGF-A*.

**Effect of** ***D. ammoniacum*** **resin-extract on embryonic angiogenesis**

Analysis of the vessels of the chick’s EEM was done to clarify the adverse effect of *D. ammoniacum *on the embryonic angiogenesis. The following steps were taken.

**Eggs**

Chicken eggs of breed Cobb (weighing 53.6±0.7 g) were acquired from Mahan Breeder Company, Kerman, Iran in which the eggs were produced under standard conditions.

**Herbal plant extract**

*D* *.* *ammoniacum* D. Don. gum resin (Cat. No. 721B) was provided from the Gyahan Darooi Co., Sirjan, Iran, in October 2015 and authenticated at the Department of Pharmacological Sciences of Kerman University, Iran. The herbal extraction was done via the Soxhlet assay for 4 hr using 100.0 g *D. ammoniacum* resin with 1000 ml of solvent (water/ethanol 80/20 v/v). The extracts were filtered and concentrated using distillation until around 20 ml of the resin remained. The crude extract solutions were obtained at 60°C or lower to remove the solvents, and completely dried in an atmospheric oven. The extraction yield was 58% (w/w), calculated per weight of the primary material.

**Herbal administration**

Fertilized eggs were incubated (55% humidity, 37.5°C) and rotated 90 degrees in incubator. On day one of the incubation period (24 hr after incubation), the wider end of eggs was disinfected by ethanol 70% and the eggshell was punctured to inoculate the herbal extract. Then, 50 μl of either *D. ammoniacum* resin-extract or sterile phosphate buffered saline was inoculated into the eggs of plant-treated and sham control groups, respectively. The eggs were re-inoculated 24 and 48 hr following the first inoculation. Fifty microliters of the extract was inoculated on the shell membrane as described previously (Oosterbaan et al., 2012; Gheorghescu et al., 2015). Groups included in this study were: group 1 (n=10): phosphate buffered saline-inoculated group (sham group); groups 2 (n=10) and 3 (n=10): herbal extract-inoculated groups, in which, the eggs were treated with herbal extract at doses of 50 or 100 mg per kg egg-weight, respectively. At Hamburger–Hamilton (HH) developmental stages 22–24 (four days after incubation), an orifice of 4 mm^2^ was created in the shell for digital capturing. The embryo Hamburger–Hamilton developmental stages was determined as described previously (Hamburger and Hamilton, 1951 ▶). The high quality images were taken using a stereomicroscope (Luxeo 4D, CA, USA) and saved as TIF files. The study was performed according to the European Ethical approaches in experimental researches (Wrigley et al., 2010 ▶). The inoculation time was chosen based on the previous reports (Thompson and Bannigan, 2007 ▶; Oosterbaan et al., 2012 ▶; Gheorghescu et al., 2015 ▶).

**Analysis of the EEM vasculature**

**Figure 1:**
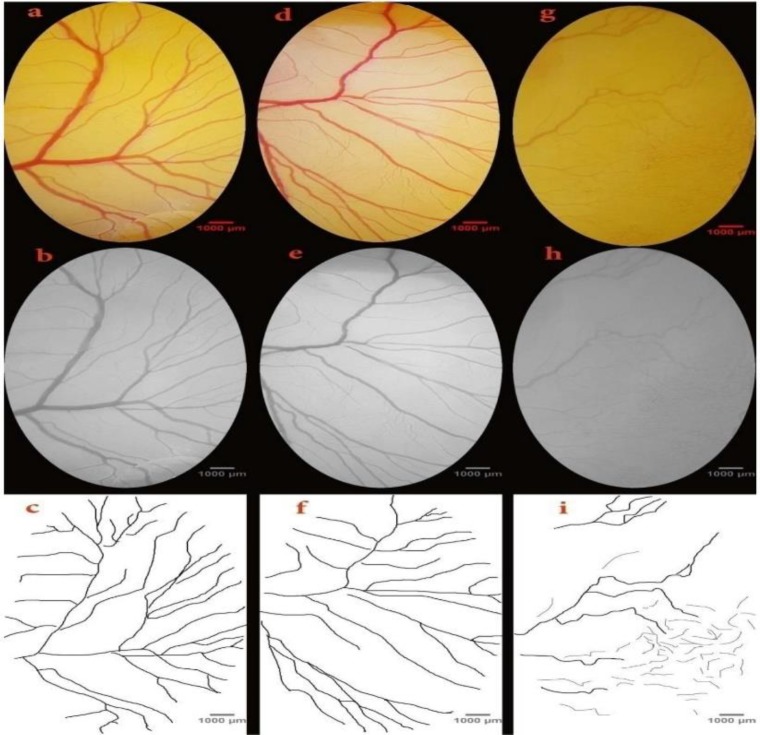
The vascular networks of the day 4 embryos are presented to illustrate the image manipulations required for the vascular branching pattern analysis. The images are captured from the embryo of the sham group (a-c) and *Dorema ammoniacum* at doses of 50 (d-f) or 100 (g-i) mg per kg egg-weight. An area of 174 mm^2^ containing 1100·×1970 pixels, was identified at the right-lateral vitelline vascular plexus (a, d, and g). The images were converted to the 8-bit format (b, e, and h). The vascular branching pattern was calculated from the skeletonized pictures (c, f, and i)

The image analyzer softwares such as ImageJ 1.48 (National Institutes of Health, USA) and MATLAB (Math works Matlab) were used for computerized analysis of the captured images. At the first step, a definite area was cleared from the images. The cleared area (174 mm^2^, 1100×1970 pixels) was determined at the right-lateral vitelline vessels (Figure 1 a, d, and g). The images were changed to the 8-bit format and manipulated to extract the schematic pattern of the vascular plexus (Figure 1 b, e, and h). Eventually, images color was changed to binary and skeletonized format (Figure 1 c, f, and i). Skeletonized format exhibits the structural aspect of the picture. The vessel pattern was analyzed for modification of parameters including vessels area, total vessels length, vascular branch, and lacunarity (Blacher et al., 2011 ▶; Magnaudeix et al., 2016 ▶). The lacunarity shows the areas without any vessel branch. In this paper, methods applied to quantify the anti-angiogenic activity of *D. ammoniacum *resin-extract, were the computerized analysis of vascular branching pattern and analysis of the mean capillary area (MCA) of the digitally acquired images from the EEM. To date, these assays have been widely used in various studies (McKay et al., 2008 ▶; Borba et al., 2016 ▶; Magnaudeix et al., 2016 ▶; Bulant et al., 2017 ▶). 

**Morphometric measurement of capillary density**

**Figure 2:**
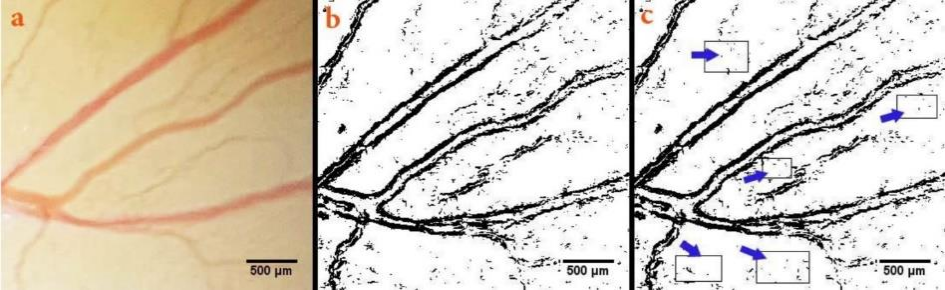
The mean capillary area (MCA) quantified from the chick’s extra-embryonic membrane on day 4 of the incubation period. Certain zone is seen inside the right-lateral vitelline vascular plexus (a). The extracted zone has been converted to a binary format (b). Five areas (arrows) without any branch vessels are selected and the percentage of the areas containing black pixels was calculated for quantification of the MCA (c). The black pixels of the image indicate the red color, or blood, in the original image

The constant zone inside the right-lateral vitelline vessels was extracted and its contrast was improved (Figure 2a). The extracted zone was converted to a binary scale (Figure 2b). From those, regions without branch vessels were chosen for analysis. Five regions were identified and their percentage including black pixels was measured (Figure 2c). The black pixels are blood in the original images. The mean of five regions are considered as the MCA (Seidlitz et al., 2004).

**Effect of** ***D. ammoniacum*** **resin-extract on the expression of** ***VEGF-A***

The relative expression of *VEGF-A* gene was determined by real-time PCR (qPCR) technique. At the first step, total RNA was extracted from the EEM vessels by the RNeasy^®^ mini kit (Qiagen, Chatsworth, CA), based on the manufacturer's protocol (n=4 in each group). The concentration of extracted RNA (ng) and the RNA purity were calculated at 260:280 nm using a spectrophotometer (NanoDrop ND-1000, Wilmington, USA). In the next step, cDNA was made using the commercial Takara kit (Takara Bio, Inc., Shiga, Japan). Then, qPCR was performed at 37°C (15 min) using 500 ng of total RNA. The SYBR Green assay (SYBR Premix Ex Taq™ II, Takara Japan) was done in the Rotorgene system (Rotor-Gene 3000 cycler machine, Corbett, Australia). The sequences of the reference gene and primers are presented in Table 1 (Gheorghescu et al., 2015 ▶). The primers amplified the 86 bp fragment of the *VEGF-A* mRNA according to the following program: 95ºC for 1 min, 40 cycles at 95ºC for 10 sec, 60ºC for 15 sec, and 72ºC for 20 sec. The *VEGF-A* expression was measured relatively to the expression of the reference gene.

**Statistical analyses**

Statistical analysis was performed using SPSS version 22. The one-way analysis of variance and Tukey's test were applied to calculate the significance of differences in the vessels parameters and *VEGF-A* expression. A p value <0.05 was determined as significant.

**Table 1:** The specific primers and reference gene sequences used for quantitative real-time RT-PCR

**Gene (** ***Gallus gallus*** **)**	**Primer Sequence (5′–3′)**		**Product size (bp)**
*VEGF-A*	Forward	CAATTGAGACCCTGGTGGAC	86
	Reverse	TCTCATCAGAGGCACACAGG	
*GAPDH*	Forward	CCTCTCTGGCAAAGTCCAAG	176
	Reverse	GGTCACGCTCCTGGAAGATA	

## Results

**Vascular branching pattern**

**Figure 3:**
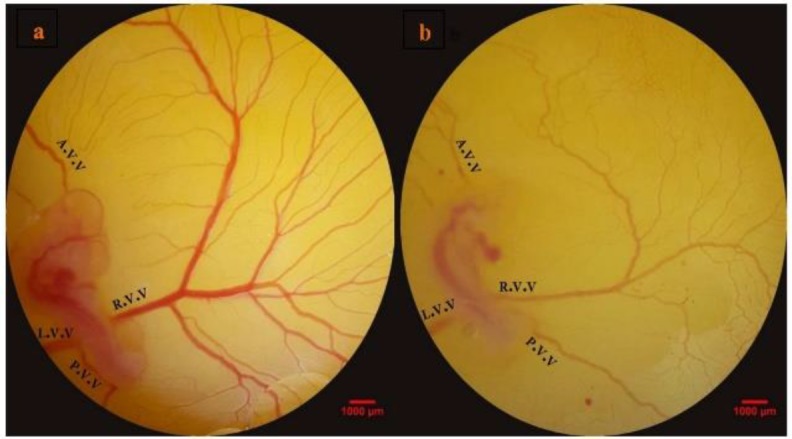
Embryonated eggs were treated three times at 24, 48 and 72 hr of the incubation period. Sham embryo with normal extra-embryonic membrane vasculature is seen (a). Embryonated egg received *Dorema ammoniacum* resin-extract at the dosage of 100 mg per kg egg-weight (b). Vascular alteration is demonstrated by the retarded vascular network. A.V.V., anterior vitelline vein; L.V.V., left lateral vitelline vessel; P.V.V., posterior vitelline vein; and R.V.V., right lateral vitelline vessel

During the imaging analysis (day 4 of embryo growth), the embryos were at stages 22–24 of HH. In the sham group, a normal plexus of vitelline vessel was seen near the embryo (Figure 3a). The blood circulated in the vessels and entered into sinus terminalis or vitelline vein. In group 3, an altered conformation of the EEM vessels was demonstrated by decreased branching (p<0.05), (Figure 3b). The analysis of vascular branching pattern following *D. ammoniacum* treatment is presented in Table 2. The herbal extract altered the vascular branching pattern in embryos treated with the highest level of the herb (p<0.05).

**Table 2:** Vascular branching pattern analysis in experimental groups

**Groups**			**Parameters**
***Dorema ammoniacum*** (**mg/kg egg-weight**)		**Sham control**	
**100**	**50**		
39.22±1.75*^+^	59.54±1.34*	63.3±1.21	**Vessels area (%)**
4722.84±2.58*^+^	8554.55±2.40*	8721.32±2.22	**Total vessels length (pixel)**
48±3.69*^+^	127±3.89*	132±4.43	**Vascular branch**
0.91±0.43*^+^	0.33±0.17*	0.33±0.05	**Lacunarity**

Values are presented as the mean ± standard error of the mean

* shows significant difference (p<0.05) compared to sham control group

+ shows significant difference (p<0.05) between *Dorema ammoniacum* at dose of 100 mg/kg compared to the *Dorema ammoniacum* at dose of 50 mg/kg

In the embryos of group 3, the parameters of the vascular network were decreased when compared with the sham group (p<0.05). In group 3, the lacunarity was increased significantly (p<0.05). The vascular pattern of the embryos in group 2 (which were inoculated with *D. ammoniacum *resin-extract at a dose of 50 mg per kg egg-weight), was similar to the sham group.

**Vessel density**

**Figure 4:**
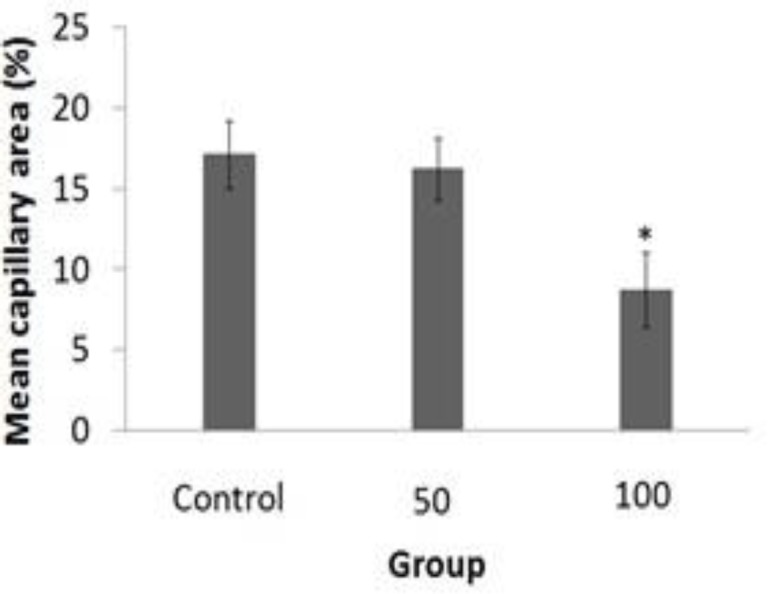
Effect of *Dorema ammoniacum* resin-extract on the early embryonic angiogenesis was assessed by mean capillary area (MCA) quantification. Data are presented for sham (n=10) and *Dorema ammoniacum* resin-extract 50 (n=10) and 100 (n=10) mg per kg egg-weight. The MCA was reduced in *D. ammoniacum*-treated group of 100 mg per kg egg-weight (error bars show standard error of mean; *p<0.05, One-Way ANOVA and *post hoc* Tukey test)

As shown in Figure 4, there was a significant decrease in MCA value of the inoculated embryos in group 3 (8.75±2.31) as compared with the sham group (17.11±2.12) (p<0.05), and group 2 (16.22±1.94) (p<0.05). Statistical analysis confirmed that embryos treated with higher dose of herbal extract showed the reduction in the MCA value (p<0.05).

**Expression of** ***VEGF-A***

**Figure 5:**
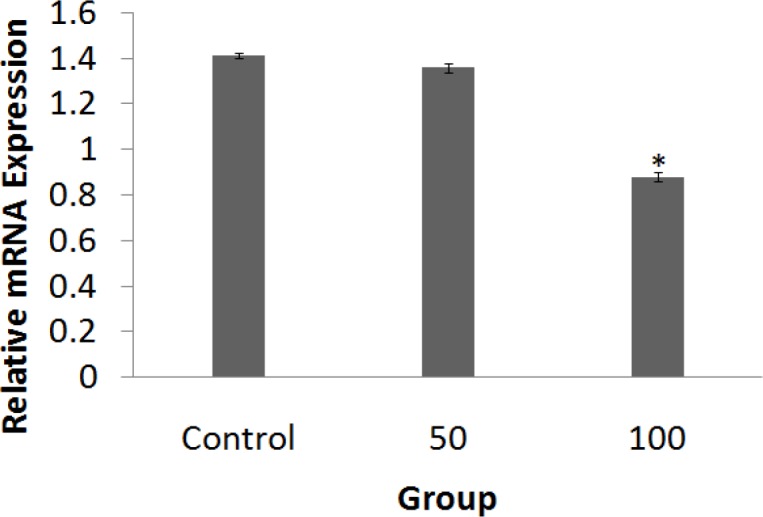
Relative mRNA expression levels of *VEGF-A* gene in experimental groups. The expression of *VEGF-A* in the chick extra-embryonic membranes (n=4 per experimental group) was decreased in the *Dorema ammoniacum*-treated group of 100 mg per kg egg-weight. *D. ammoniacum* resin-extract was administered at doses of 50 or 100 mg per kg egg-weight (error bars show standard error of mean; *p<0.05, One-Way ANOVA and *post hoc* Tukey test)

The *VEGF-A* expression level was assessed using qPCR on the 4^th^ day of the growing period. The expression level of *VEGF-A* decreased significantly in the treated embryos of group 3 compared to group 2 (p<0.05) and sham group embryos (p<0.05) (Figure 5).

## Discussion

Today, in developing societies, herbal products are being used for preventive and therapeutic purposes. However, determination of the side effects of medicinal plants in human still needs further investigations. Hence, some aspects of the embryonic vascular toxicity including a reduction in vessels area, total vessels length, and vascular branch as well as an increase in lacunarity of the *D. ammoniacum* were evaluated in the present study using the chick’s EEM model. The chick’s EEM model was used by various researchers for evaluating the embryo toxicity of agents (Majidian-Eydgahi et al., 2015 ▶; Araghi et al., 2016 ▶; Paradkar et al., 2017 ▶). The anti-angiogenic property of *D. ammoniacum* in embryo has not been fully described. Based on our experiment, it is suggested that *D. ammoniacum* can cause vascular injury at the doses equal or greater than 100 mg per kg. It would be useful to assess the vascular toxicity of the *D. ammoniacum* in more details in the future investigations. Vascular alteration might be due to the cytotoxic activity of *D. ammoniacum *(Yousefzadi et al., 2011 ▶). In addition, the specific inherent properties of *D. ammoniacum* could be joined with its adverse activity. For example, some researches confirmed that different species of *Dorema* have a genotoxic activity that causes damage to DNA (Eskandani et al., 2014 ▶).

Alteration in gene expression, as well as vascular development, may provide an association between *D. ammoniacum *inoculation and gestational defects of the fetus. *D. ammoniacum* at dose of 100 mg per kg egg-weight seems to induce adverse effect on gene expression pattern. The decrease of vascular branch and angiogenesis, induced by *D. ammoniacum*, may limit flow of blood stream through the vessels. Alterations in blood flow can cause a reduction in shear stress, which is received by the endothelium (Groenendijk et al., 2005 ▶). Generally, when shear stress increases, *VEGF-A* is up-regulated (Resnick et al., 2003 ▶); therefore, reduction of shear stress after *D. ammoniacum *inoculation might decrease *VEGF-A* expression.

To the best of our knowledge, this is the first study investigating the early anti-angiogenic property of *D. ammoniacum* resin-extract using a chick’s EEM model. Our findings coincide well with previous studies focused on the side effect and toxicity of herbs to the vascular system. Moreover, our data indicated that *D. ammoniacum *not only induces a negative effect on the early vascular development but also alters the gene expression. We suggest that these alterations may result in devastating consequences in the fetus and this phenomenon require further evaluation.

In conclusion,* D. ammoniacum* applied to the chick’s EEM was vasculo-toxic at the doses equal to or higher than 100 mg/kg egg weight; therefore, its consumption should be limited in pregnancy, particularly in industrialized societies that face an increasing tendency on the use of herbs. In the current study, we also employed a successful chick’s EEM model that offers a hopeful assay for investigation of the toxicity of different herbs during gestational period in which, the appraisement cannot be performed in fetus. 
